# Characterisation of a rare, reassortant human G10P[14] rotavirus strain detected in Honduras

**DOI:** 10.1590/0074-02760170083

**Published:** 2018-01

**Authors:** Osbourne Quaye, Sunando Roy, Kunchala Rungsrisuriyachai, Mathew D Esona, Ziqian Xu, Ka Ian Tam, Dina J Castro Banegas, Gloria Rey-Benito, Michael D Bowen

**Affiliations:** 1 Centers for Disease Control and Prevention, Gastroenteritis and Respiratory Viruses Laboratory Branch, Atlanta, Georgia, USA; 2 University of Ghana, Department of Biochemistry, Cell and Molecular Biology, West African Center for Cell Biology of Infectious Pathogens, Legon, Accra, Ghana; 3 China Center for Disease Control and Prevention, National Institute for Viral Disease Control and Prevention, Beijing, China; 4 Nacional Colonia La Campaña, Tegucigalpa, Honduras; 5 Pan American Health Organization, Washington DC, USA

**Keywords:** rotavirus, viral genome, viral proteins, non-structural proteins, reassortant

## Abstract

**BACKGROUND:**

Although first detected in animals, the rare rotavirus strain G10P[14] has been sporadically detected in humans in Slovenia, Thailand, United Kingdom and Australia among other countries. Earlier studies suggest that the strains found in humans resulted from interspecies transmission and reassortment between human and bovine rotavirus strains.

**OBJECTIVES:**

In this study, a G10P[14] rotavirus genotype detected in a human stool sample in Honduras during the 2010-2011 rotavirus season, from an unvaccinated 30-month old boy who reported at the hospital with severe diarrhea and vomiting, was characterised to determine the possible evolutionary origin of the rare strain.

**METHODS:**

For the sample detected as G10P[14], 10% suspension was prepared and used for RNA extraction and sequence independent amplification. The amplicons were sequenced by next-generation sequencing using the Illumina MiSeq 150 paired end method. The sequence reads were analysed using CLC Genomics Workbench 6.0 and phylogenetic trees were constructed using PhyML version 3.0.

**FINDINGS:**

The next generation sequencing and phylogenetic analyses of the 11-segmented genome of the G10P[14] strain allowed classification as G10-P[14]-I2-R2-C2-M2-A3-N2-T6-E2-H3. Six of the genes (VP1, VP2, VP3, VP6, NSP2 and NSP4) were DS-1-like. NSP1 and NSP5 were AU-1-like and NSP3 was T6, which suggests that multiple reassortment events occurred in the evolution of the strain. The phylogenetic analyses and genetic distance calculations showed that the VP7, VP4, VP6, VP1, VP3, NSP1, NSP3 and NSP4 genes clustered predominantly with bovine strains. NSP2 and VP2 genes were most closely related to simian and human strains, respectively, and NSP5 was most closely related to a rhesus strain.

**MAIN CONCLUSIONS:**

The genetic characterisation of the G10P[14] strain from Honduras suggests that its genome resulted from multiple reassortment events which were possibly mediated through interspecies transmissions.

Group A rotaviruses have been a major cause of severe diarrhea worldwide among children younger than five years (Parashar et al. 2006) and account for a global mortality of approximately 215,000 annually (Tate et al. 2016). Rotaviruses belong to the *Reoviridae* family of viruses. The viral genome of rotavirus, which is composed of 11 double-stranded RNA segments and encapsulated in a concentric triple-layered protein structure, encodes for six structural and five or six non-structural proteins. A binomial classification system has been used traditionally for genotyping rotaviruses based on antigenic or genetic characterisation of the outer capsid proteins VP7 (G-type) and VP4 (P-type). However, a classification system which makes use of the open reading frame sequences of all the genes is currently widely used as the standard nomenclature (Matthijnssens et al. 2008). The viral proteins and non-structural proteins in the order VP7-VP4-VP6-VP1-VP2-VP3-NSP1-NSP2-NSP3-NSP4-NSP5 are represented by the genotypes Gx-P[x]-Ix-Rx-Cx-Mx-Ax-Nx-Tx-Ex-Hx, respectively. At least 35 G-types, 50 P-types, 26 I-types, 21 R-types, 19 C-types, 19 M-types, 30 A-types, 20 N-types, 21 T-types, 26 E-types, and 21 H-types have been detected to date (https://rega.kuleuven.be/cev/viralmetagenomics/virus-classification/newgenotypes). With respect to the G and P genotypes in humans globally, G1-4, G9, P[8] and P[4] are still the most common; and the most frequently detected strain combinations include G1P[8], G2P[4], G3P[8], G4P[8] and G9P[8] (Doro et al. 2014). Rotaviruses have also been classified into Wa-like, DS-1-like and AU-1-like genogroups based on cross hybridisation studies (Nakagomi et al. 1989). The genogroups classification was also later shown in genomic studies (Matthijnssens & Van Ranst 2012).

In developing countries, detection of uncommon strains and unusual genotype combinations are more frequent than in developed ones, but are still a rare event compared to the detection of the common human genotypes (Bourdett-Stanziola et al. 2010, Seheri et al. 2014). Latin America is one region in the developing world where many countries have introduced rotavirus vaccination as part of their national immunisation programs (Desai et al. 2011). Studies from Latin American countries suggest that the epidemiology of rotavirus in the region is the same as the global epidemiology, with more than 70% of strains detected being those that are most frequently detected globally (Castello et al. 2004, de Oliveira et al. 2009). There are a significant number of mixed infections and non-typeable strains, but unusual G-P combinations such as G1P[6], G1P[9], G2P[6], G3P[6], G2P[8], G4P[4], G5P[6], G9P[4], G4P[14],G10P[8] and G11P[6] are less frequently, observed (Linhares et al. 2011, Quaye et al. 2013). Some of these uncommon strains were locally dominant and associated with severe disease (Araujo et al. 2007).

Until 2013, all reports of the rare G10P[14] strains detected in humans were either on the VP7 and VP4 genes characterisations, or the VP6 and NSP4 genes in addition to the VP7 and VP4 genes (Ghosh et al. 2007, Steyer et al. 2010), and thus far, the G10P[14] strain has not been detected in the Americas, making this study the first report in the region.

## MATERIALS AND METHODS

Stool samples collected during the 2010-2011 rotavirus season in Honduras (n = 50) that had tested positive for rotavirus antigen by enzyme immunoassay (EIA) were sent to the Rotavirus Surveillance Laboratory at the US Centers for Disease Control and Prevention for EIA confirmation, genotyping, and nucleotide sequencing. Ethical approval was obtained from the National Health Services of Honduras. For all the samples, 10% stool suspensions were prepared from the specimens using PBS, and the presence of the rotavirus antigen was confirmed by EIA using the Premier™ Rotaclone® Detection Kit (Meridian Diagnostics, Inc., Cincinnati, OH). The immunoassays were read spectrophotometrically at 450 nm on an MRX Revelation plate reader (Dynex Magellan Biosciences, Chantilly VA). Immunoassays with absorbance values greater than 0.15 were considered positive for rotavirus antigen.

Following the manufacturer's protocols for initial characterisation, rotavirus double-stranded RNA was extracted from the 10% fecal suspensions using the automated KingFisher extraction system (Thermo Fisher Scientific, Waltham, MA) with the Max 96 Viral RNA Isolation Kit (Ambion, Inc., Austin, TX). The extracted RNAs were used as templates for reverse transcription polymerase chain reaction (RT-PCR), genotyping, and nucleotide sequencing as previously described (Hull et al. 2011) to identify the VP4 and VP7 genotypes. The sequence for each gene was compared to rotavirus sequences in the nr/nt database using the BLASTN program at the National Center for Biotechnology Information website (http://www.ncbi.nlm.gov/BLAST/).

For a sample that was detected as G10P[14] from an unvaccinated 30-month old boy with acute gastroenteritis, large volumes of the 10% stool suspension were prepared and used for RNA extraction and sequence independent amplification as recently described (Potgieter et al. 2009, Jere et al. 2011) to amplify all the 11 gene segments. The amplicons were sequenced by next-generation sequencing using the Illumina MiSeq 150 paired end method (Genomics Lab, Hudson Alpha Institute for Biotechnology, Huntsville, Alabama).

Illumina sequence reads were analysed using CLC Genomics Workbench 6.0. A combination of *de novo* assembly and subsequent mapping to reference strain was used to obtain the full-length genome of the strain. The assembled gene sequences were aligned with reference rotavirus gene sequences using the ClustalW program within MEGA 5.05 package (Tamura et al. 2011). Once aligned, the optimal evolutionary model that best fit each sequence dataset was identified using AICc criterion implemented in jModeltest2. The best models identified were TIM3+I (NSP1), TPM2uf+G (NSP2), TIM2+G (NSP3), TPM2uf+I (NSP4), TrN+I+G (NSP5, VP6), TIM2+I (VP1), GTR+I (VP2, VP3), GTR+G (VP4), and TIM3+I+G (VP7). Phylogenetic trees were constructed using PhyML version 3.0 with aLRT statistics computed for estimation of branch support (Guindon et al. 2010), and p-distances were computed in MEGA 5.05 to determine the similarities of the genes to reference strains in GenBank. The scale bars on the phylogenetic trees represent proportion of substitution on a branch of each tree. The gene sequences were submitted to RotaC (http://rotac.regatools.be/) for genotype assignments. All the gene sequences of the G10P[14] genome has been deposited into the GenBank sequence database under accession numbers KU956006 through KU956016.

## RESULTS

The G10P[14] strain, which was detected in a stool sample collected from an unvaccinated 30-month old boy who reported at the hospital with severe diarrhea and vomiting in the Distrito Central of the Department of Francisco Morazan in Honduras during the 2010-2011 rotavirus season, was amplified by sequence independent amplification and sequenced by Illumina next-generation sequencing technology. The sequence amplification products for the VP1-VP3 were not visible by agarose gel electrophoresis, even though the amplification was successful, and resulted in thousands of reads when sequenced on the Illumina platform. The submission of the sequences to RotaC resulted in assignment of a G10-P[14]-I2-R2-C2-M2-A3-N2-T6-E2-H3 constellation, showing that six of the genes (VP6, VP1, VP2, VP3, NSP2 and NSP4) were DS-1-like, NSP1 and NSP5 were AU-1-like, and NSP3 was the T6 genotype. The results suggest the occurrence of multiple reassortment events in the evolution of the strain. Figure (A-K) shows the phylogenetic trees for all the 11 genes and Table I shows the percentage similarity of the genes to the most closely related reference strains in Genbank. The phylogenetic analyses and genetic p-distance calculations showed that the VP7 gene of the strain from Honduras clustered in a clade that contains bovine, equine, ovine, and human strains [Figure (A)]. The VP4 gene is closest to the bovine strain Sun9, whereas the VP6 gene is closest to bovine UK-tc strain and the human strain ITA-tc/PA169 in a mixed clade of human and bovine strains [Table I, Figure (B-C)]. The VP6 gene occupied a basal position to the lineage that contains human and bovine reference strains including closely-related RotaTeq® strains [Figure (C)]. VP1 is closely related to RotaTeq® strains and a human G6P[14] strain from Australia [Table I, Figure (D)], VP2 is most closely related to human strain A549 [Table I, Figure (E)], and NSP2 is most closely related to a simian RRV strain [Table I, Figure (H)]. The NSP5 gene was closest to a Rhesus tissue culture strain RTRV [Table I, Figure (K)]. The VP3 gene belongs to a predominantly bovine clade, the NSP1 gene is in a clade with human and bovine strains, the NSP3 gene is in a clade with mixed strains, and the NSP4 gene belongs to a clade with bovine and equine strains as shown in Figure (F, G, I, J), respectively, and Table I. While it is a common phenomenon in such studies for genes to be closely related to reference strains with a percent similarity of over 99%, it was interesting to note that the percent similarities of the most closely related strains in GenBank to all the respective genes of the G10P[14] strain from Honduras were less than 99%. Table II is a comparison of the genome constellation of the G10P[14] strain from Honduras with other G10P[14] strains that have been deposited in GenBank.

**(A-K):**
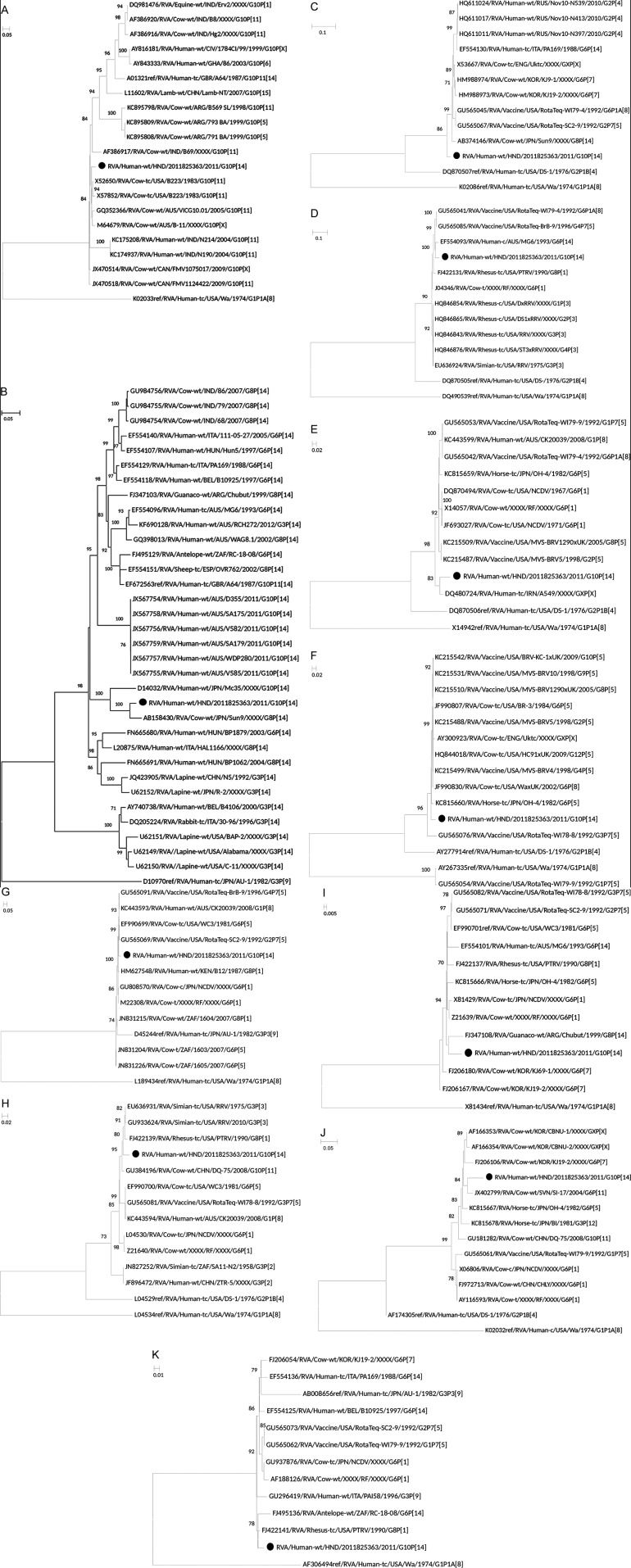
maximum likelihood phylograms indicating the genetic relationships of nucleotide sequences of VP7 (A), VP4 (B), VP6 (C), VP1 (D), VP2 (E), VP3 (F), NSP1 (G), NSP2 (H), NSP3 (I), NSP4 (J) and NSP5 (K) of human G10P[14] rotavirus strain from Honduras (labeled with filled circles), with sequences of human and animal rotavirus strains from the GenBank database. The trees were drawn to scale, with the scale bars representing proportion of substitution on a branch of each phylogenetic tree. Only aLRT values ≥ 70% are shown.

**TABLE I:** Percentage similarity of gene segments for Honduras G10P[14] rotavirus strain to genetically most closely related GenBank reference strains

Gene segment for	Genotype	Cut-off values	*% Similarity of genes to GenBank strains (Origin/Name/Strain) (Accession #)
VP7	G10	80	96.2 (Bovine/B223/GxP[x]) (X52650)
VP4	P [14]	80	95.1 (Bovine/Su n 9/G8P[14]) (AB158430)
VP6	I2	85	95.8 (Bovine/ U K-tc/Gx P[x]) (X53667)
VP1	R2	83	96.4 (RotaTeq/W179-4/G6P[8]) (GU565041)
VP2	C2	84	94.6 (Human/A549/GxP[x]) (DQ480724)
VP3	M2	81	97.0 (Bovine/MVS-BRV4/G4P[5]) (KC215499)
NSP1	A3	79	96.6 (Bovine/Cow-tc WC3/G6P[5]) (EF990699)
NSP2	N2	85	97.5 (Simian/RRV/G3P[3]) (EU636931)
NSP3	T6	85	96.7 (Bovine/RF/GxP[x]) (Z21639)
NSP4	E2	85	94.7 (Bovine/Cow-wt SVN/G6P[11]) (JX402799)
NSP5	H3	91	98.7 (Rhesus/tc-RTRV/G8P[1]) (FJ422142)

* the percent similarities were based on the nucleotide distances of the Honduras strain to GenBank strains.

**TABLE II:** Comparison of the genomes of G10P[14] rotavirus strains deposited in GenBank

Rotavirus strain	VP7	VP4	VP6	VP1	VP2	VP3	NSP1	NSP2	NSP3	NSP4	NSP5	Reference
RVA/Human-wt/HON/5363/2011/G10P[14]	G10	P[14]	I2	R2	C2	M2	A3	N2	T6	E2	H3	Strain in this paper
RVA/Human-wt/AUS/V585/2011/G10P[14]	G10	P[14]	I2	R2	C2	M2	A11	N2	T6	E2	H3	Cowley etal. (2013)
RVA/Human-wt/AUS/D355/2011/G10P[14]	G10	P[14]	I2	R2	C2	M2	A11	N2	T6	E2	H3	″
RVA/Human-wt/AUS/SA175/2011/G10P[14]	G10	P[14]	I2	R2	C2	M2	A11	N2	T6	E2	H3	″
RVA/Human-wt/AUS/SA179/2011/G10P[14]	G10	P[14]	I2	R2	C2	M2	A11	N2	T6	E2	H3	″
RVA/Human-wt/AUS/V582/2011/G10P[14]	G10	P[14]	I2	R2	C2	M2	A11	N2	T6	E2	H3	″
RVA/Human-wt/AUS/WDP280/2011/G10P[14]	G10	P[14]	I2	R2	C2	M2	A11	N2	T6	E2	H3	″
RVA/Human-wt/ITA/PR457/2009/G10P[14]	G10	P[14]	I2	R2	C2	M2	A11	N2	T6	E2	H3	Medici etal. (2015)
RVA/Human-tc/GBR/A64/1987/G10P11[14]	G10	P[14]	I2	R2	C2	M1	A3	N2	T1	E2	H3	Heiman et al. (2008)
RVA/Human-wt/IND/KOL-29/2014/G10P[14]	G10	P[14]	I2	R2	C2	M2	A11	N2	T6	E2	H3	Mandal et al. (2016)
RVA/Human-wt/IND/KOL-383/2014/G10P[14]	G10	P[14]	I2	R2	C2	M2	A11	N2	T6	E2	H3	″
RVA/Human-wt/VNM/NT0082/2007/G10P[14]	G10	P[14]	I2	R2	C2	M2	A3	N2	T6	E2	H3	Do etal. (2017)
RVA/Cow-wt/IND/RUBV81/2006/G10P[14]	G10	P[14]	I2	Rx	*Cx	*Mx	*Ax	*Nx	Tx	E2	H3	Ghosh et al. (2007)
RVA/Human-wt/SVN/SI-R241/07/2007/G10P[14]	G10	P[14]	I2	Rx	*Cx	*Mx	*Ax	*Nx	Tx	E2	*Hx	Steyer et al. (2010)
RVA/Human-wt/SVN/SI-R240/07/2007/G10P[14]	G10	P[14]	I2	Rx	*Cx	*Mx	*Ax	*Nx	Tx	E2	*Hx	″
RVA/Human-wt/SVN/SI-R230/07/2007/G10P[14]	G10	P[14]	I2	Rx	*Cx	*Mx	*Ax	*Nx	Tx	E2	*Hx	″
RVA/Human-wt/SVN/SI-R128/07/2007/G10P[14]	G10	P[14]	12	Rx	*Cx	*Mx	*Ax	*Nx	Tx	E2	*Hx	″
RVA/Human-wt/SVN/SI-2393/07/2007/G10P[14]	G10	P[14]	12	Rx	*Cx	*Mx	*Ax	*Nx	Tx	E2	*Hx	″

* the genes that are labelled x (instead of numbers) were not available in GenBank.

## DISCUSSION

The detection of rare rotavirus strains is a cause for concern due to the implications that these uncommon strains may have for existing, and yet to be developed, rotavirus vaccines, as to whether the strains will be protected against vaccine-primed immunity. Due to the segmented genome of the virus, different combinations of the segments could be obtained from reassortment and result in the formation of new genome constellations (Doro et al. 2015). The detection of such strains during hospital-based rotavirus surveillance programs, suggest that rare strains are also able to cause disease. Since first reported in animals 2009, the G10P[14] rotavirus genotype has not been detected frequently (Varshney et al. 2002, Matthijnssens et al. 2009). Previous reports of the G10P[14] strain have linked the origins of the uncommon genotype to animals, such as bovines, of the Mammalian order Artiodactyla (Ghosh et al. 2007, Steyer et al. 2010, Cowley et al. 2013, Medici et al. 2015). The most common human rotavirus genotypes in the Latin American region, including Honduras, are G1P[8], G9P[8], G2P[4], and recently G9P[4] (de Oliveira et al. 2009, Linhares et al. 2011, Quaye et al. 2013).

The genome constellation of the G10P[14] rotavirus strain from Honduras, which was characterised in this study by whole genome sequencing, suggests the occurrence of interspecies transmission activities since the virus contains DS-1-like (genogroup 2), AU-1-like (genogroup 3) strains, and a T6 genome segment coding for the non-structural protein that facilitates translation (NSP3). Strains with DS-1-like signatures are thought to be derived from bovine strains, whereas AU-1-like strains are thought to be originated from dogs and cats (Yamamoto et al. 2011, Doan et al. 2015). Previous characterisation of G10P[14] strains from Slovenia suggested at least two-horizontal interspecies transmission events that originated through a bovine-human interaction (Steyer et al. 2010). The VP7, VP6 and NSP4 genes in the Slovenian G10P[14] strains were closely related to the corresponding genes for strains that have been found in bovines. The genome could have been formed during a co-infection of a host with different strains of rotaviruses.

The Honduras G10P[14] strain is different in gene constellation from the other human G10P[14] strains that have been submitted to GenBank. The recently reported wild-type G10P[14] rotavirus strains from Vietnam (Do et al. 2017) and a tissue culture strain from Great Britain (Heiman et al. 2008) have a genomic constellation that is similar to the G10P[14] from Honduras (Table II). The strains from Vietnam, Great Britain and Honduras has the A3 genotype whereas other strains from other parts of the world including Australia (Cowley et al. 2013), India (Mandal et al. 2016), Italy (Medici et al. 2015) has the A11 genotype for the gene that codes for the interferon antagonising non-structural protein, NSP1 (Table II). The observed percent similarities of less than 99% for the relatedness of genes obtained in this study to GenBank reference strains suggest accumulation of mutations across all the 11 genes of the Honduras strain (Table I). To the best of our knowledge, G10 and P[14] strains combination have not been identified in Honduras. However, G10 strains associated with P[9] (Linhares et al. 2011), and P[14] strains associated with G4 (Tam et al. 2014) have been reported in the Latin American region. The G10P[14] strain from Honduras when compared to other G10P[14] strains suggests that the uncommon genotype is novel. However the report is limited by the fact that there is not that much information of genomic data on rotaviruses from animals, and to also deduce if the Honduras strain was formed from strains that are present within the Americas or were introduced into Latin America from other parts of the world.

The emergence of uncommon rotavirus strains, such as the G10P[14] strain herein characterised, may have a negative implication for vaccine efficacy and effectiveness. Even though the licensed vaccines have been effective against rotavirus strains that were originally not targets, the effectiveness cannot be guaranteed for all strains, especially the ones that are formed from inter-species transmissions. Rotavirus vaccination has been introduced into the national immunisation programs in multiple countries of Latin America and resulted in significant decreases in childhood diarrhea morbidity and mortality (Desai et al. 2011). The introduction of the rotavirus vaccine in Honduras in 2009 has yielded a reduction in hospitalisation and mortality by 11-20% (de Oliveira et al. 2013) compared to observations made in relatively more developed countries which had reductions in the range of 70-90% (Cortese et al. 2013, Payne et al. 2013). The difference in reduction could be attributable to the presence of rotavirus strains for which the vaccine introduced is not as effective.

In summary, the results of the whole genome characterisation of a G10P[14] rotavirus strain from Honduras is consistent with the detection of an uncommon genotype with a novel genome constellation. This is the first report of a human G10P[14] strain from the Latin American region, even though G10 and P[14] strains associated with other VP4 and VP7 genotypes, respectively, have been detected. The occurrence of the rare strain with gene segments that have animal origins suggest interspecies transmission of rotavirus strains and may have resulted from a co-infection of a common host. The continuous detection of uncommon strains in the post vaccine introduction era emphasizes the need for monitoring the emergence of new rotavirus strains.
